# Telebehavioral Health for Caregivers of Children With Behavioral Health Needs to Address Caregiver Strain: Cohort Study

**DOI:** 10.2196/59475

**Published:** 2024-08-26

**Authors:** Theoren Loo, Myra Altman, David Grodberg, Jennifer La Guardia, Dena Bravata

**Affiliations:** 1 Brightline Palo Alto, CA United States; 2 Clinical Excellence Research Center Stanford University Palo Alto, CA United States; 3 School of Medicine Yale University New Haven, CT United States; 4 Center for Primary Care and Outcomes Research Stanford University Palo Alto, CA United States

**Keywords:** adolescent, child, caregiver, family health, resilience, psychological, mental health, pediatric, pediatrics, paediatric, paediatrics, children, youth, adolescents, teen, teens, teenager, teenagers, strain, burden, caregiving, caregivers, carer, carers, informal care, family care, spousal care, telehealth, telemedicine, technology-enabled, mobile phone

## Abstract

**Background:**

Behavioral health conditions among children have worsened over the past decade. Caregivers for children with behavioral health conditions are at risk for two types of caregiver strain: (1) an objective strain, that results directly from the child’s condition and (2) subjective strain, that arises from the caregiver’s feelings regarding these events.

**Objective:**

This study aimed to evaluate the impact of a technology-enabled pediatric and family behavioral health service on caregivers’ strain among a commercially insured population. We also explore the common symptom clusters of caregiver strain to better understand the caregiver presentation to inform future care planning.

**Methods:**

We examined changes in caregiver strain using the Caregiver Strain Questionnaire-Short Form 7 over the course of their child’s web-based behavioral health care between 2021 and 2023 using a pre-post study design. Common caregiver strain symptom clusters were identified using Ward hierarchical agglomerative clustering.

**Results:**

The majority of children were White 60.8% (1002/1647), female 53.6% (882/1647), and aged between 5 and 9 years (33.7%, 555/1647). Families fall broadly into 4 groups based on what drives caregiver strain the most, namely those experiencing (1) disrupted family relationships and time interruption, (2) missed work, (3) worried about their child’s future and feeling tired and sad, and (4) financial strain. Caregiver strain, which was associated with the child’s disease severity (*P*<.001), decreased significantly in all therapeutic groups.

**Conclusions:**

Web-based family-oriented behavioral health care can improve caregiver strain and reduce family and time disruptions, missed work, and financial strain. Sources of caregiver strain vary and may be overlooked when relying on the conventional scoring of the Caregiver Strain Questionnaire-Short Form 7.

## Introduction

Over the past decade, the prevalence of behavioral health conditions among children has steadily worsened [1,2]. Currently, 17% of children aged 2 to 8 years are diagnosed with a mental health condition [3] and 37% of adolescents aged 12 to 17 years report persistent feelings of sadness or hopelessness [1,4]. The adults who care for these children are at risk for 2 types of caregiver strain, that are (1) an objective strain, that results directly from the child’s condition (eg, disrupted family relationships, financial strain, and interruptions at work) and (2) subjective strain, that arises from the caregiver’s feelings regarding these events (eg, guilt, anger, and sadness) [5]. Although the severity of the child’s problems is often the best predictor of caregiver strain, poor access to convenient, affordable in-network providers is a common source of caregiver strain, even among commercially insured families [5]. Given evidence that reducing parental strain can improve the effectiveness of evidence-based treatments for children with mental illness [6,7], a robust understanding of the sources of caregiver strain may lead to more beneficial approaches for the whole family. While previous studies have evaluated caregiver strain in terms of internalizing and externalizing domains [8], specific sources of strain have not been documented.

The costs for outpatient behavioral care for commercially insured populations increased by 84% between 2018 and 2021 compared with an increase of 60% in overall behavioral health costs during that interval [9]. Self-insured employers pay a disproportionate share of these costs because they provide direct payment for mental health services, and bear indirect costs, such as reduced employee productive work time [10]. In response to the increased prevalence of mental health concerns and these escalating costs, approximately 80% of self-insured employers report implementing behavioral health benefits, including employee assistance programs and others that increase access to low-cost mental health services, including telemental health [9]. However, telehealth use among children and teenagers lags behind adults, despite recent telehealth expansions for behavioral health services [11].

In response to these trends, we implemented a comprehensive, technology-enabled pediatric and family behavioral health service for commercially insured populations. The intervention provides access to educational content, telebehavioral health coaching, and web-based psychotherapy and medication management. Children are triaged to either telebehavioral health coaching, telepsychotherapy, or telepsychiatry based on their clinical acuity.

In this study, we hypothesize that a telebehavioral health service is effective in reducing strain among parents caring for a child with a mental health condition. We also explore the common symptom clusters of caregiver strain to better understand the caregiver presentation to inform future care planning. In a secondary analysis, we evaluate the relationship between caregiver strain and child behavioral health severity, which were strongly correlated in a previous study [5]. We hope that this study will provide important observations around remediable symptoms and patterns of caregiver strain that will inform the development of telebehavioral health offerings for children and their families.

## Methods

### Overview

This study followed the Strengthening the Reporting of Observational Studies in Epidemiology (STROBE) reporting guideline for cohort studies [12]. STROBE guidelines are used in observational studies to improve the quality, transparency, integrity, and reproducibility of scientific literature.

### Intervention

Brightline is a technology-enabled behavioral health care platform for children (aged 18 months to 17 years) and their families [13,14]. Briefly, the web-based platform is provided to commercially insured populations in all 50 states through their employer-sponsored health benefits. Families have the option to receive care through a computer or smartphone. There are 4 main intervention offerings available, that are (1) psychoeducational content, (2) telebehavioral health coaching, (3) telepsychotherapy, and (4) telepsychiatry. A previous study from Brightline showed that 75% of children in coaching and 69% of children in telepsychotherapy and telepsychiatry demonstrated reliable improvement in their psychosocial functioning [14]. All families using the service have access to a rich library of scientifically informed educational content, including videos and exercises on a wide range of topics, including modules for children and their caregivers (eg, parent management training [PMT]). Families also have access to coaching support through chat.

Children and their caregivers, with preclinical or subclinical concerns, have access to a protocolized, evidence-informed 1:1 telecoaching program. Coaching programs typically last 4 to 6 sessions beginning with a structured assessment of the child’s needs followed by either caregiver- or child-caregiver dyad–focused coaching. For example, caregiver skills training often includes understanding how to identify and focus on positive behaviors (as opposed to focusing on the child’s negative behaviors), understanding and demonstrating effectiveness in using praise and other types of rewards to reinforce the child’s positive behavior, and setting up behavioral plans and troubleshooting their implementation—modules developed based on best practices of PMT [15].

Families with children with higher acuity needs are referred to licensed therapists for evidence-based, family-centric telepsychotherapy. Interventions are derived from evidence-based approaches, including cognitive behavioral therapy, components of dialectical behavior therapy, and caregiver-mediated interventions (eg, PMT), rooted in trauma-informed and culturally responsive approaches to care. Standardized pathway-driven approaches, such as the Modular Approach to Therapy for Anxiety, Depression, Trauma, or Conduct Problems model (MATCH-ADTC), are used to support how care is implemented [16]. Children and teens who indicate plans to harm themselves or others have required admission to a psychiatric hospital or residential treatment facility within the previous 30 days, complicating substance use, or severely disordered eating are externally referred for immediate care and related care coordination.

In some instances, where the child can appropriately receive care from more than 1 type of program, families are able to enroll in a program of their preference.

Including a caregiver in care is standard. The focus of interventions differs based on age and presenting concerns, such that either a PMT (caregiver-only within sessions) or a dyadic care model (caregiver and child sessions; caregiver-only sessions are included) are used. In PMT, the child remains the focus, but the caregiver attends sessions alone, and the focus is on strategies to reduce negative behaviors in the home and improve positive connections between caregivers and their child. In dyadic care, the child and caregiver both participate in the course of care.

### Participants

We included child-caregiver dyads who enrolled in the intervention between October 2021 and July 2023. Dyads must have completed at least 1 coaching, psychotherapy, or psychiatry session and completed a baseline and follow-up assessment. If participants’ needs change as they progress through care and a different level of care is indicated, they may move to a different program. For this study, we exclude dyads that participated in more than 1 program.

### Data Collected

Within each dyad, caregivers completed validated, self-reported assessments to evaluate the clinical effectiveness of receiving telebehavioral health coaching, telepsychotherapy, and telepsychiatry services. Baseline assessments were collected on the web before engagement with coaches and psychotherapists, and follow-up assessments were collected every 4 weeks during care [17].

#### Caregiver Strain

Caregivers completed the Caregiver Strain Questionnaire-Short Form 7 (CGSQ-SF7) [18], a caregiver-completed assessment of objective and subjective caregiver strain experienced over the past month. Potential causes of caregiver strain are rated on a scale from 1 (not at all) to 5 (very much). A total of 4 items assessing interruption of personal time, missing work, financial strain, and disruption of familial relationships due to the child’s current challenges comprise the CGSQ-SF7 objective strain subscale, and 3 items assessing their own experience of sadness, worry, and fatigue or strain as a result of their child’s challenges make up the CGSQ-SF7 subjective strain subscale. Subscale scores are calculated by taking the average of the item ratings. The CGSQ-SF7 total score is determined by adding the subscale scores together. The reliability and validity of the CGSQ-SF7 objective and subjective strain subscales are comparable to the full-length CGSQ [18].

#### Pediatric Symptoms

Caregivers completed the Pediatric Symptom Checklist-17 (PSC-17). The PSC-17 is a caregiver-completed survey that measures a child’s total psychosocial functioning, with subscales that measure function in the areas of internalizing, attention, and externalizing concerns [19].

### Analyses

For this retrospective cohort analysis, a pre-post study design was used based on CGSQ-SF7 assessments collected at baseline and discharge. If dyads were lost to follow-up, last-value-carried-forward imputation was implemented.

We separated the dyads into 2 groups, those with high caregiver strain and those with nonelevated caregiver strain. High strain was defined as having either a CGSQ-SF7 total score of 7.0 or more, objective score of 3.0 or more, or subjective score of 4.0 or more at baseline, and caregivers that did not meet any of these criteria were categorized as having nonelevated strain [20]. Counts of caregivers with reliable improvement were determined among dyads with high caregiver strain. Reliable improvement was defined as having a minimum detectable change of 1.13 or more on the total score, 0.66 or more for the objective score, or 0.76 or more for the subjective score [20]. Counts of caregivers that achieved maintenance were calculated for dyads with nonelevated strain at baseline. Maintenance was defined as having a minimum detectable change no less than –1.13 on the total score, –0.66 for the objective score, and –0.76 for the subjective score [20].

To evaluate patterns of symptom clustering among those with high caregiver strain at baseline, we used Ward hierarchical agglomerative clustering with Euclidean distances. Euclidean distance is the length between 2 points in a multidimensional space and is used to measure similarity among objects in cluster analyses [21]. Hierarchical agglomerative clustering is an unsupervised machine learning algorithm that progressively aggregates objects based on the distance metric and linkage criterion [22] and has been used for symptom clustering in previous studies [23]. Paired, 2-tailed *t* tests were used to evaluate changes in scores from baseline to follow-up across symptom clusters and CGSQ-SF7 subscales among caregivers with high strain at baseline. Cohen *d* was used to measure the effect size within each grouping.

To evaluate the relationship between caregiver strain and child behavioral health severity, we performed univariate linear regressions to test the association between CGSQ-SF7 and PSC-17 scores at baseline and the change in CGSQ-SF7 and PSC-17 scores from baseline to follow-up.

All statistical analyses were performed in R software (version 4.1.2; R Foundation for Statistical Computing).

### Ethical Considerations

This study was classified as exempt from consent requirements under human subjects review by the Western Institutional Review Board (WIRB)-Copernicus Group Institutional Review Board, per Federal Regulations for the Protection of Human Research Subjects (45CFR 46.104(d)(4)) (protocol Brightline.004) [24].

## Results

### Participants

Demographics of the children are reported in Table 1. Of the 1647 children, 806 (48.9%) completed a course of care in coaching, 750 (45.5%) in psychotherapy, and 91 (5.5%) in psychiatry. Dyads, including teenagers (aged 13-17 years), made up the largest proportion of children in psychiatry and psychotherapy (56%, 51/91 and 39.1%, 293/750), compared with those who received coaching (23.1%, 186/806). The majority of children in the dyads were White (60.8%, 1002/1647); however, 12.8% (210/1647) reported identifying as multiracial. There were no differences in baseline CGSQ-SF7 scores by race of the child (Table 2). Overall, 882 (53.6%) of children in dyads were female. Notably, the proportion of females was larger than males in psychiatry and psychotherapy (63.7%, 58/91 and 57.1%, 428/749) compared with those who received coaching (49.1%, 396/806). On average, dyads completed 6.7 (SD 4.3) sessions in coaching, 10.6 (SD 6.8) sessions in psychotherapy, and 19.2 (SD 11.7) sessions in psychiatry.

**Table 1:** Demographic characteristics of children in the study.

Demographics			Total (N=1647)		Children in coaching (n=806)		Children in psychotherapy (n=750)		Children in psychiatry (n=91)
**Race, n (%)**									
	White	1002 (60.8)		465 (57.7)		484 (64.5)		53 (58.2)	
	Multiracial	210 (12.8)		115 (14.3)		85 (11.3)		10 (11.0)	
	Hispanic	146 (8.9)		63 (7.8)		65 (8.7)		18 (19.8)	
	Black	99 (6.0)		53 (6.6)		43 (5.7)		3 (3.3)	
	Asian	97 (5.9)		56 (7.0)		36 (4.8)		5 (5.5)	
	Prefer not to say	47 (2.9)		24 (3.0)		22 (2.9)		1 (1.1)	
	Other	40 (2.4)		26 (3.2)		14 (1.9)		—^a^	
**Sex, n (%)**									
	Female	882 (53.6)		396 (49.1)		428 (57.1)		58 (63.7)	
	Male	764 (46.4)		410 (50.9)		321 (42.8)		33 (36.3)	
**Age at enrollment (years)**, **n (%)**									
	<5	73 (4.4)		71 (8.8)		2 (0.3)		—	
	5-9	555 (33.7)		315 (39.1)		220 (29.3)		20 (22.0)	
	10-12	489 (29.7)		234 (29.0)		235 (31.3)		20 (22.0)	
	13-17	530 (32.2)		186 (23.1)		293 (39.1)		51 (56.0)	
Completed sessions, mean (SD)			9.2 (6.8)		6.7 (4.3)		10.6 (6.8)		19.2 (11.7)
Baseline PSC-17^b^ total score, mean (SD)			14.2 (5.6)		13.2 (5.5)		14.8 (5.7)		15.8 (5.1)

^a^Not applicable.

^b^PSC-17: Pediatric Symptom Checklist-17 is a caregiver-completed survey that measures a child’s total psychosocial functioning.

**Table 2:** Baseline caregiver strain score (Caregiver Strain Questionnaire-Short Form 7 total) by child race, gender, and age.

Demographics		Coaching		Psychotherapy		Psychiatry	
		Mean (SD)	*P* value	Mean (SD)	*P* value	Mean (SD)	*P* value
**Race**			.30		.96		.46
	White	5.1 (1.6)		5.5 (1.7)		5.8 (1.7)	
	Multiracial	4.8 (1.5)		5.6 (1.4)		5.7 (1.3)	
	Hispanic	4.7 (1.8)		5.4 (1.9)		6.3 (2.1)	
	Black	5.0 (2.0)		5.4 (1.5)		6.9 (1.7)	
	Asian	5.0 (1.8)		5.5 (1.8)		4.9 (.8)	
**Sex**			.01		.01		.01
	Female	4.8 (1.6)		5.4 (1.7)		5.5 (1.7)	
	Male	5.2 (1.7)		5.7 (1.7)		6.5 (1.6)	
**Age at enrollment (years)**			.01		.01		.09
	<5	5.7 (1.8)		3.8 (0.24)		—^a^	
	5-9	5.0 (1.6)		5.8 (1.7)		6.5 (1.3)	
	10-12	5.0 (1.7)		5.6 (1.6)		6.0 (1.9)	
	13-17	4.8 (1.7)		5.3 (1.8)		5.5 (1.8)	

^a^Not applicable.

### Caregiver Strain Symptom Clusters

**Figure 1:**
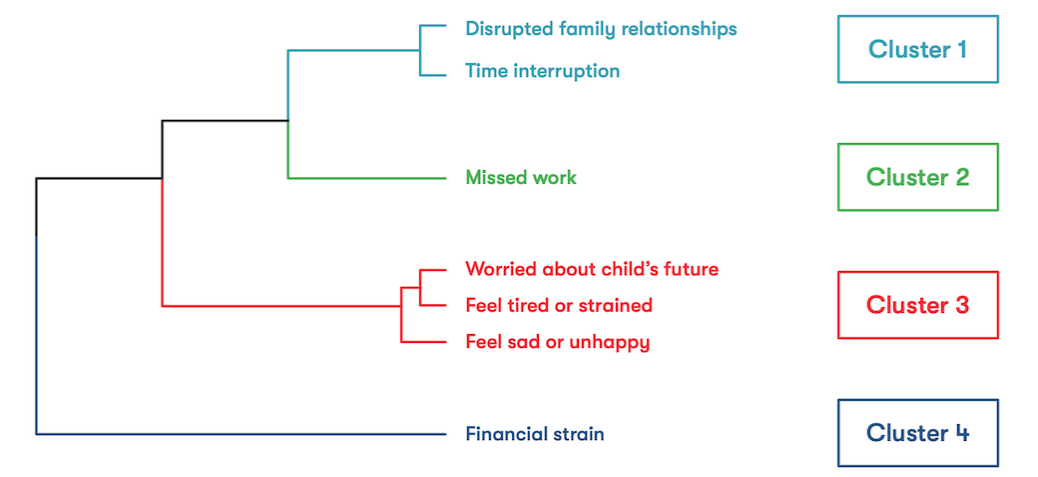
Caregiver strain symptom clusters among caregivers with a high Caregiver Strain Questionnaire-Short Form 7 at baseline. This figure shows the key drivers of strain for caregivers of children with mental illness.

The structure of the dendrogram produced from the hierarchical agglomerative clustering procedure suggests that four symptom clusters fit the data well (Figure 1), with key drivers of caregiver strain being (1) disrupted family relationships and time interruption, (2) missed work, (3) worry about their child’s future and who felt tired and sad, and (4) financial strain. At baseline, 71.4% (1176/1647) of caregivers experienced symptoms in Cluster 1, 61.0% (1004/1647) experienced symptoms in Cluster 2, 80.4% (1325/1647) experienced symptoms in Cluster 3, and 20.8% (342/1647) experienced symptoms in Cluster 4.

The Euclidean distance at which the symptoms merged for Cluster 3 was small and aligned with the grouping of symptoms defined in the CGSQ-SF7 subjective strain subscale. Therefore, further analysis of Cluster 3 was not conducted. However, the financial strain and missed work symptoms merged with other clusters at large Euclidean distances, suggesting that these symptoms are distinct from each other. Consequently, we treated the financial strain and work symptoms as independent clusters. This observation is a departure from the CGSQ-SF7 objective strain subscale grouping that clusters financial strain, missed work, time interruption, and disrupted family relationship symptoms together [18].

### Caregiver Strain

Overall, the highest baseline caregiver strain was in the group receiving psychiatry and lowest in the group receiving coaching (Table 3). Baseline subjective measures of caregiver strain were higher in all groups than objective measures of caregiver strain (Table 3). Among those with high strain, a reliable improvement in the CGSQ-SF7 total was demonstrated in 78.9% (90/114) caregivers in coaching, 68.5% (113/165) caregivers in psychotherapy, and 65.2% (15/23) caregivers in psychiatry (Table 4). Maintenance on the CGSQ-SF7 total was achieved among caregivers without high strain for (93.2% (645/692) caregivers in coaching, 90.4% (529/585) of caregivers in psychotherapy, and 83.8% (57/68) of caregivers in psychiatry (Table 4). Rates of reliable improvement and maintenance were high for CGSQ-SF7 objective and subjective scores (Table 4). CGSQ-SF7 total, subscale, and cluster scores decreased from baseline to follow-up across all programs (Table 5).

**Table 3:** Caregivers with high and low-to-moderate Caregiver Strain Questionnaire-Short Form 7 scores at baseline.

Child-enrolled program	Total				Objective				Subjective		
	Baseline, mean (SD)	High strain^a^, n (%)	Low-to-moderate strain^b^, n (%)	Baseline, mean (SD)		High strain^a^, n (%)	Low-to-moderate strain^b^, n (%)	Baseline, mean (SD)		High strain^a^, n (%)	Low-to-moderate strain^b^, n (%)
Coaching	5.0 (1.7)	114 (14.1)	692 (85.9)	2.1 (0.8)		127 (15.8)	679 (84.2)	2.9 (1.0)		171 (21.2)	635 (78.8)
Psychotherapy	5.5 (1.7)	165 (22.0)	585 (78.0)	2.3 (0.8)		180 (24.0)	570 (76.0)	3.2 (1.0)		235 (31.3)	515 (68.7)
Psychiatry	5.8 (1.7)	23 (25.3)	68 (74.7)	2.4 (0.9)		25 (27.5)	66 (72.5)	3.4 (1.0)		37 (40.7)	54 (59.3)

^a^High strain was defined by having a total score of 7.0 or more, an objective score of 3.0 or more, or a subjective score of 4.0 or more at baseline.

^b^Low-to-moderate strain was defined as having a total score less than 7.0, an objective score less than 3.0, or a subjective score less than 4.0.

**Table 4:** Rates of reliable improvement and maintenance among caregivers on the Caregiver Strain Questionnaire-Short Form 7.

Child-enrolled program	Total		Objective		Subjective	
	Reliable improvement^a^, n (%)	Maintained^b^, n (%)	Reliable improvement^a^, n (%)	Maintained^b^, n (%)	Reliable improvement^a^, n (%)	Maintained^b^, n (%)
Coaching	90 (78.9)	645 (93.2)	93 (73.2)	633 (93.2)	119 (69.6)	596 (93.9)
Psychotherapy	113 (68.5)	529 (90.4)	114 (63.3)	506 (88.8)	160 (63.8)	477 (92.6)
Psychiatry	15 (65.2)	57 (83.8)	17 (68.0)	58 (87.9)	18 (48.6)	48 (88.9)

^a^Reliable improvement was determined among caregiver-child dyads with high strain and defined as having a minimum detectable change of −1.13 or lower on the total score, −0.66 for the objective score, and −0.76 for the subjective score.

^b^Maintained was determined among caregiver-child dyads with nonelevated strain and defined as having a minimum detectable change no less than −1.13 on the total score, −0.66 for the objective score, and −0.76 for the subjective score.

**Table 5:** Caregiver Strain Questionnaire-Short Form 7 total, subscales, and cluster scores among caregivers at baseline and follow-up.

Child-enrolled program		Baseline, mean (SD)	Follow-up, mean (SD)	Paired mean difference (95% CI)	Percent change (%)	*t* test (*df*)	*P* value^a^	Cohen *d*
**Coaching**								
	Total	7.8 (0.8)	5.3 (1.6)	2.5 (2.2-2.8)	–32.1	16.8 (113)	<.001	1.9
	Objective	7.5 (1)	5.3 (1.7)	2.2 (1.9-2.5)	–29.3	14.9 (126)	<.001	1.6
	Subjective and Cluster 3^b^	7.3 (1)	5.1 (1.6)	2.2 (1.9-2.4)	–30.1	17.0 (170)	<.001	1.6
	Cluster 1^c^	6.4 (1.7)	5.5 (1.5)	0.9 (0.7-1.1)	–14.0	10.3 (409)	<.001	0.6
	Cluster 2^d^	2.7 (0.8)	1.8 (0.9)	0.9 (0.7-0.9)	–33.3	17.2 (445)	<.001	0.9
	Cluster 4^e^	2.7 (0.9)	1.7 (0.9)	1.0 (0.8-1.2)	–37.0	9.4 (130)	<.001	1.1
**Psychotherapy**								
	Total	7.8 (0.6)	5.8 (1.9)	2.0 (1.7-2.3)	−25.6	13.7 (164)	<.001	1.4
	Objective	7.5 (0.9)	5.7 (1.9)	1.8 (1.5-2.1)	−24.0	13.1 (179)	<.001	1.2
	Subjective and Cluster 3^b^	7.3 (0.9)	5.5 (1.9)	1.8 (1.6-2.0)	−24.7	15.2 (234)	<.001	1.2
	Cluster 1^c^	6.7 (1.6)	5.8 (1.7)	1.0 (0.8-1.2)	−13.4	10.2 (373)	<.001	0.7
	Cluster 2^d^	2.7 (0.8)	2.0 (1.1)	0.7 (0.6-0.8)	−25.9	15.0 (491)	<.001	0.8
	Cluster 4^e^	2.7 (0.9)	2.0 (1.1)	0.7 (0.5-0.9)	−25.9	7.5 (186)	<.001	0.7
**Psychiatry**								
	Total	8.1 (0.8)	6.1 (1.8)	2.0 (1.3-2.7)	−24.7	5.9 (22)	< .001	1.2
	Objective	7.9 (1.0)	6.3 (1.7)	1.6 (0.8-2.2)	−20.3	4.5 (24)	<.001	1.1
	Subjective and Cluster 3^b^	7.4 (1.1)	5.7 (1.8)	1.7 (1.2-2.3)	−23.0	6.4 (36)	<.001	1.1
	Cluster 1^c^	6.8 (1.6)	6.0 (1.7)	0.8 (0.3-1.3)	−11.8	3.3 (54)	<.001	0.5
	Cluster 2^d^	2.9 (0.9)	2.2 (1.1)	0.7 (0.5-1.1)	−24.1	5.5 (65)	<.001	0.8
	Cluster 4^e^	2.8 (1.0)	1.9 (0.8)	0.9 (0.5-1.4)	−32.1	4.1 (23)	<.001	1.0

^a^*t* test *P* value is significant at .008 after adjusting for multiple comparisons using Bonferroni correction.

^b^Cluster 3 is made up of the same items as the Caregiver Strain Questionnaire-Short Form 7 subjective subscale.

^c^Cluster 1 includes disrupted family relationships and time interruption items.

^d^Cluster 2 includes the missed work item.

^e^Cluster 4 includes the financial strain item.

### Association Between Caregiver Strain and Child Behavioral Health Severity

There was a strong positive association between baseline CGSQ-SF7 total score and PSC-17 total score (coaching: β=.16, *P*<.001; psychotherapy: β=.13, *P*<.001; and psychiatry: β=.14, *P*<.001; Table S1 in Multimedia Appendix 1). There was also a strong positive association between the change in CGSQ-SF7 total score and PSC-17 total score from baseline to follow-up (coaching: β=.14, *P*<.001; psychotherapy: β=.13, *P*<.001; and psychiatry: β=.09, *P*=.01; Table S1 in Multimedia Appendix 2).

## Discussion

### Principal Findings

Although there is a growing literature supporting digital mental health models for the adult population [25-27], there is far less research evaluating specific digital mental health models for children, teens, and their families [28]. In this study, we evaluated the effect of a web-based, multitiered pediatric mental health care intervention on caregiver strain and identified symptom clusters of caregiver strain measured within the CGSQ-SF7 associated with caring for a child with a mental health condition.

This analysis has 2 key findings. First, overall caregiver strain as measured by the CGSQ-SF7 total score, which is highly associated with their child’s symptom severity, can improve with a mix of clinical interventions for the child and caregiver including coaching, psychotherapy, and psychiatry delivered on the web. Our results were consistent with previous studies that demonstrated that the severity of the child’s problems is a key predictor of caregiver strain [29]. By matching patients and their families to the appropriate care pathway (ie, coaching vs psychotherapy vs psychiatry) based on both the child’s symptom severity and family preference, this study suggests that these sources of caregiver strain can be improved. Not surprisingly, given their baseline acuity, families in coaching required fewer sessions and could be cared for in a shorter timeframe than families receiving the most intensive psychiatric care, which suggests that lower-intensity interventions may be an important part of the overall care landscape for families. Although other authors have found caregiver strain to vary by race and ethnicity in the adult mental health literature [6], we did not. This would be worth evaluating in a future analysis of caregivers for children.

Second, the drivers of caregiver strain measured within the CGSQ-SF7 vary among families with children in need of behavioral health services. While providing care focused on the child is beneficial for caregiver outcomes, it may also be valuable to target additional care to the specific needs of the caregiver. Targeted interventions may be more possible with the identification of key drivers of caregiver strain, which may be overlooked when relying on the CGSQ-SF7 total, objective, or subjective subscales alone. Specifically, disrupted family relationships and time interruptions, missing work, and financial strain are distinct caregiver strain symptoms within the CGSQ-SF7 that independently clustered in our population. Despite these symptoms being objective measures of caregiver strain, it may be insightful for providers to not only administer the CGSQ-SF7, but also to individually evaluate these distinct symptom clusters within the CGSQ-SF7 objective subscale, and include these considerations in care planning, as they may provide insights into the specific action plan and supportive interventions for each family. For example, for those caregivers for whom disrupted family relationships and time interruption were key drivers of caregiver strain, whole-family counseling or marital counseling may be warranted [30]. Similarly, those caregivers most worried about their child’s future who feel tired and sad may benefit from individual care or respite programs [31]. In addition, employers may have a role in mitigating strain among caregivers of children with mental illness. For caregivers for whom missed work was a key driver of strain, a more flexible work schedule may be helpful, and a robust benefit offering of no and low-cost mental health solutions may be particularly helpful for caregivers for whom the financial strain of caring for their children was the key driver of their strain. Future research should specifically evaluate additional interventions for caregivers, and whether those are most impactful delivered separately for caregivers or embedded within care for the family.

### Limitations

Our analysis had several limitations; the first being our retrospective, observational design that did not include randomization or a control group. The second limitation is driven by our limited demographic data on each dyad which only included the age, gender, and race and ethnicity of the child. This lack of broad demographic data meant we were unable to control other important demographic variables, such as socioeconomic status, rurality, and family structure, since users are not currently required to provide this information to receive care. Third, while uncommon, it is possible for multiple caregivers to be involved in care. Fourth, we do not account for the number of care sessions completed by each dyad and how dose may moderate outcome estimates. Finally, this analysis aggregated all mental health diagnoses and concerns into one group. Specific diagnoses and concerns may have varying outcomes to web-based behavioral health programs and have different sources of caregiver strain. Future studies will address these limitations.

### Conclusion

This analysis of a web-based, multi-tiered pediatric mental health care model demonstrates a positive impact on caregiver strain among a privately insured population with access to a computer or smartphone. We also identified common sources of caregiver strain measured within the CGSQ-SF7 among parents caring for a child with a mental health condition. We hope this work will inform the ongoing development of web-based pediatric mental health models and the designs of health benefits for commercially insured populations.

## Appendix

Multimedia Appendix 1 Univariate linear regressions examining the relationship between Caregiver Strain Questionnaire (CGSQ-SF7 Total score) and Pediatric Symptom Checklist (PSC-17 Total score).

Multimedia Appendix 2 Univariate linear regressions examining the relationship between the change in Caregiver Strain Questionnaire (CGSQ-SF7 Total score) and Pediatric Symptom Checklist (PSC-17 Total score) from baseline to follow-up.

## Abbreviations

CGSQ-SF7: Caregiver Strain Questionnaire-Short Form 7
MATCH-ADTC: Modular Approach to Therapy for Anxiety, Depression, Trauma, or Conduct Problems
PMT: parent management training
PSC-17: Pediatric Symptom Checklist-17
STROBE: Strengthening the Reporting of Observational Studies in Epidemiology

## Data Availability

The data sets generated during and/or analyzed during this study are not publicly available but are available from the corresponding author on reasonable request. STROBE guidelines were adhered to and submitted as Multimedia Appendices.
