# Supplementary material for: Telebehavioral Health for Caregivers of Children With Behavioral Health Needs to Address Caregiver Strain: Cohort Study
Source: JMIR Pediatr Parent. 2024 Aug 26;7:e59475. doi: 10.2196/59475 (PMC11384170; doi:10.2196/59475)
Supplement: Multimedia Appendix 1 [file pediatrics_v7i1e59475_app1.docx]

Multimedia Appendix 1. Univariate linear regressions examining the relationship between Caregiver Strain Questionnaire (CGSQ-SF7 Total score) and Pediatric Symptom Checklist (PSC-17 Total score).

Table S1

| **Child enrolled program** | **β** | **Standard error** | **T value** | ***P* value** | ***R*^2^** |
| --- | --- | --- | --- | --- | --- |
| **Coaching** | .16 | .14 | 19.30 | <.001 | 0.28 |
| **Psychotherapy** | .13 | .01 | 13.99 | <.001 | 0.21 |
| **Psychiatry** | .14 | .03 | 4.13 | <.001 | 0.15 |
