# Supplementary material for: Telebehavioral Health for Caregivers of Children With Behavioral Health Needs to Address Caregiver Strain: Cohort Study
Source: JMIR Pediatr Parent. 2024 Aug 26;7:e59475. doi: 10.2196/59475 (PMC11384170; doi:10.2196/59475)
Supplement: Multimedia Appendix 2 [file pediatrics_v7i1e59475_app2.docx]

**Multimedia Appendix 2.** Univariate linear regressions examining the relationship between the change in Caregiver Strain Questionnaire (CGSQ-SF7 Total score) and Pediatric Symptom Checklist (PSC-17 Total score) from baseline to follow-up.

**Table S1.**

| **Child enrolled program** | **β** | **Standard error** | **T value** | ***P* value** | ***R*^2^** |
| --- | --- | --- | --- | --- | --- |
| **Coaching** | .14 | .01 | 11.2 | <.001 | 0.18 |
| **Psychotherapy** | .13 | .01 | 12.8 | <.001 | 0.18 |
| **Psychiatry** | .09 | .03 | 2.8 | .01 | 0.08 |
